# Supplementary material for: SMAC Mimetics Synergistically Cooperate with HDAC Inhibitors Enhancing TNF-α Autocrine Signaling
Source: Cancers (Basel). 2023 Feb 18;15(4):1315. doi: 10.3390/cancers15041315 (PMC9954505; doi:10.3390/cancers15041315)

Supplemental Figure 1

Figure 2A whole blot

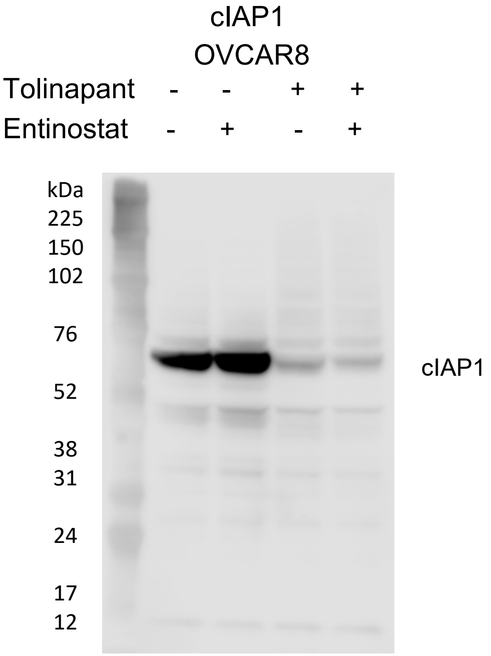

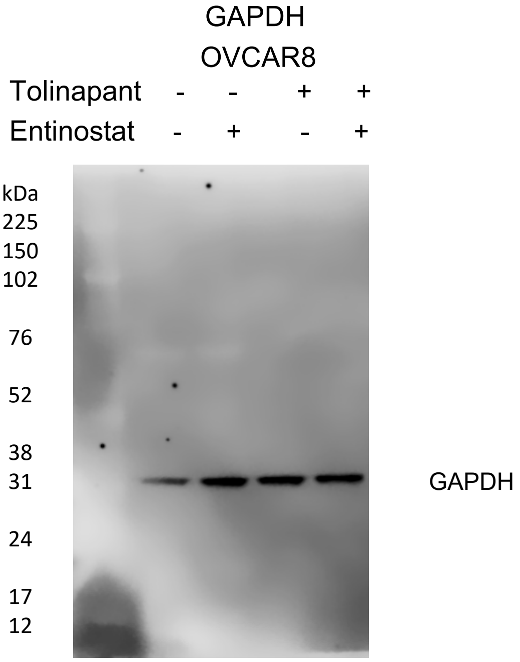

Supplemental Figure 2

Figure 2B whole blot

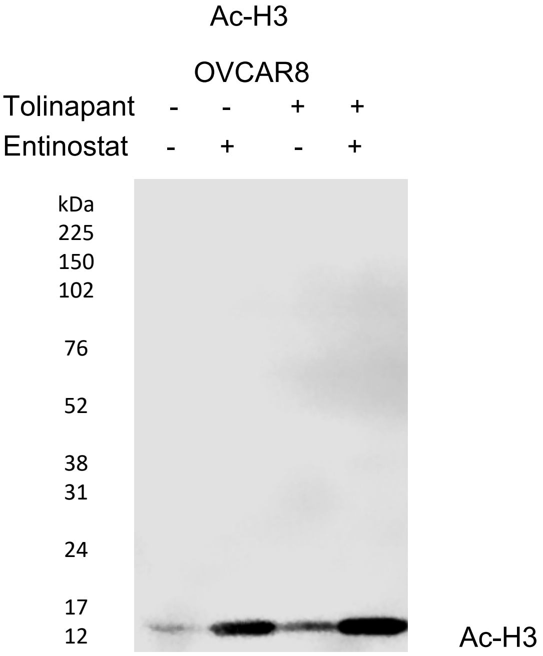

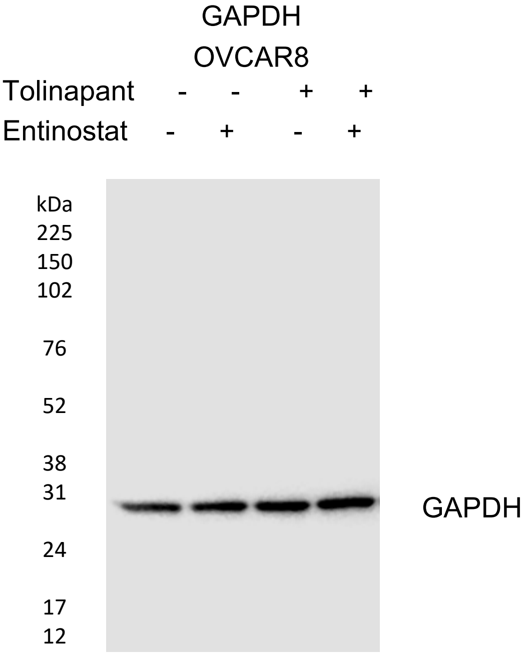

Supplemental Figure 3

Figure 2C WCL
XIAP whole blot

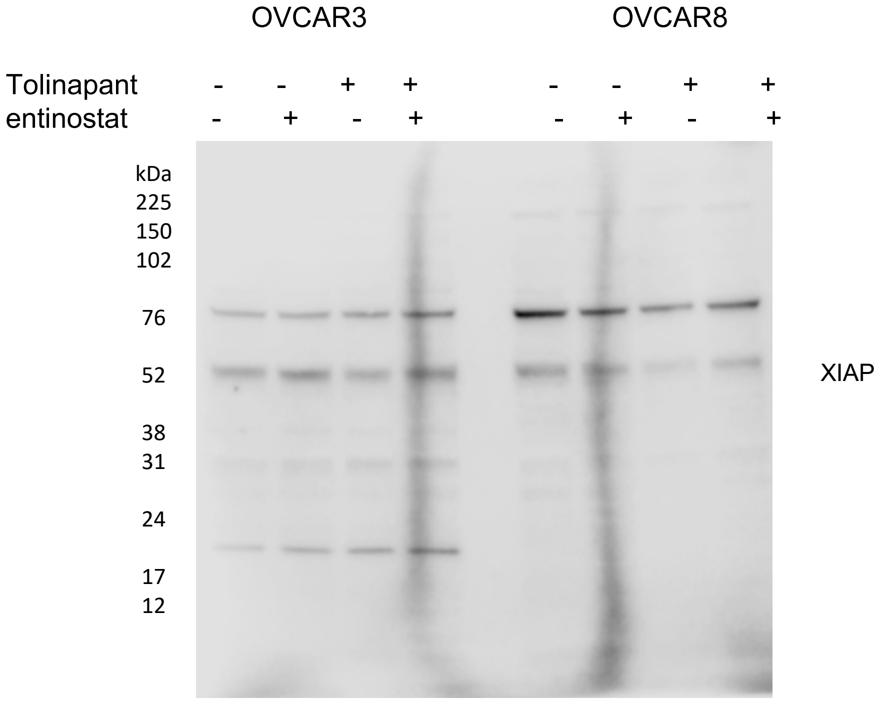

Supplemental Figure 4

Figure 2C WCL
SMAC whole blot

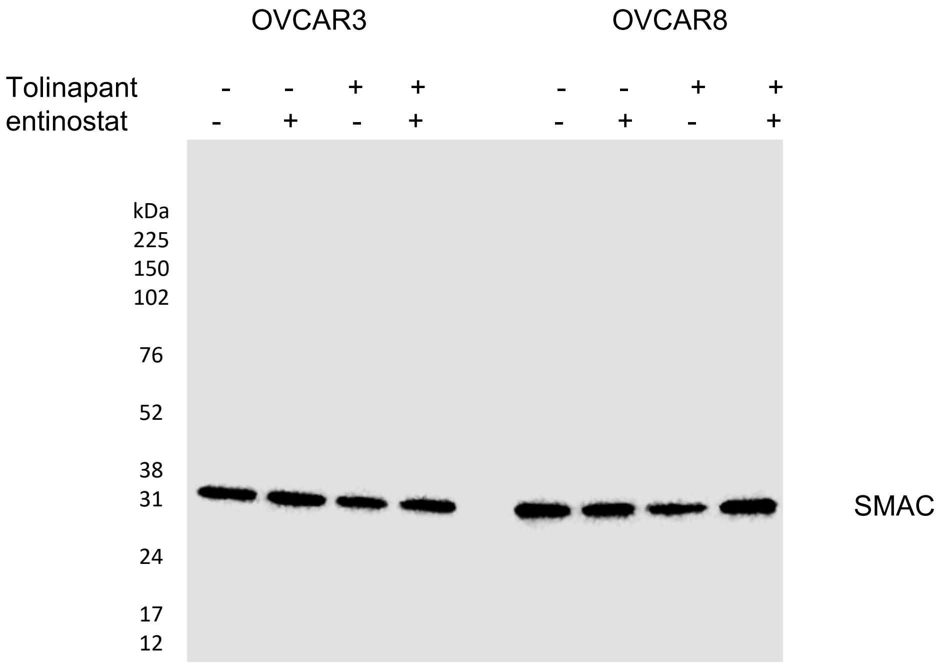

Supplemental Figure 5

Figure 2C WCL
GAPDH whole blot

OVCAR3 OVCAR8

Tolinapant	-	-	+	+	-	-	+	+
entinostat	-	+	-	+	-	+	-	+

kDa
225
150
102
76
52
38
31
24
17
12

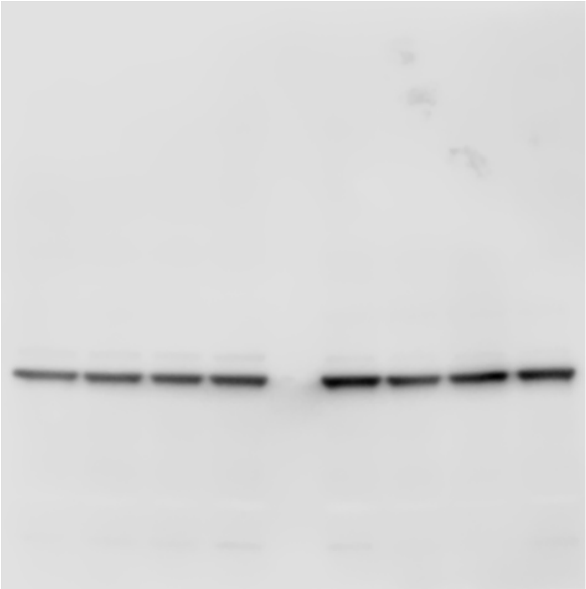

GAPDH

Supplemental Figure 6

Figure 2C IP-XIAP
XIAP, SMAC whole blot

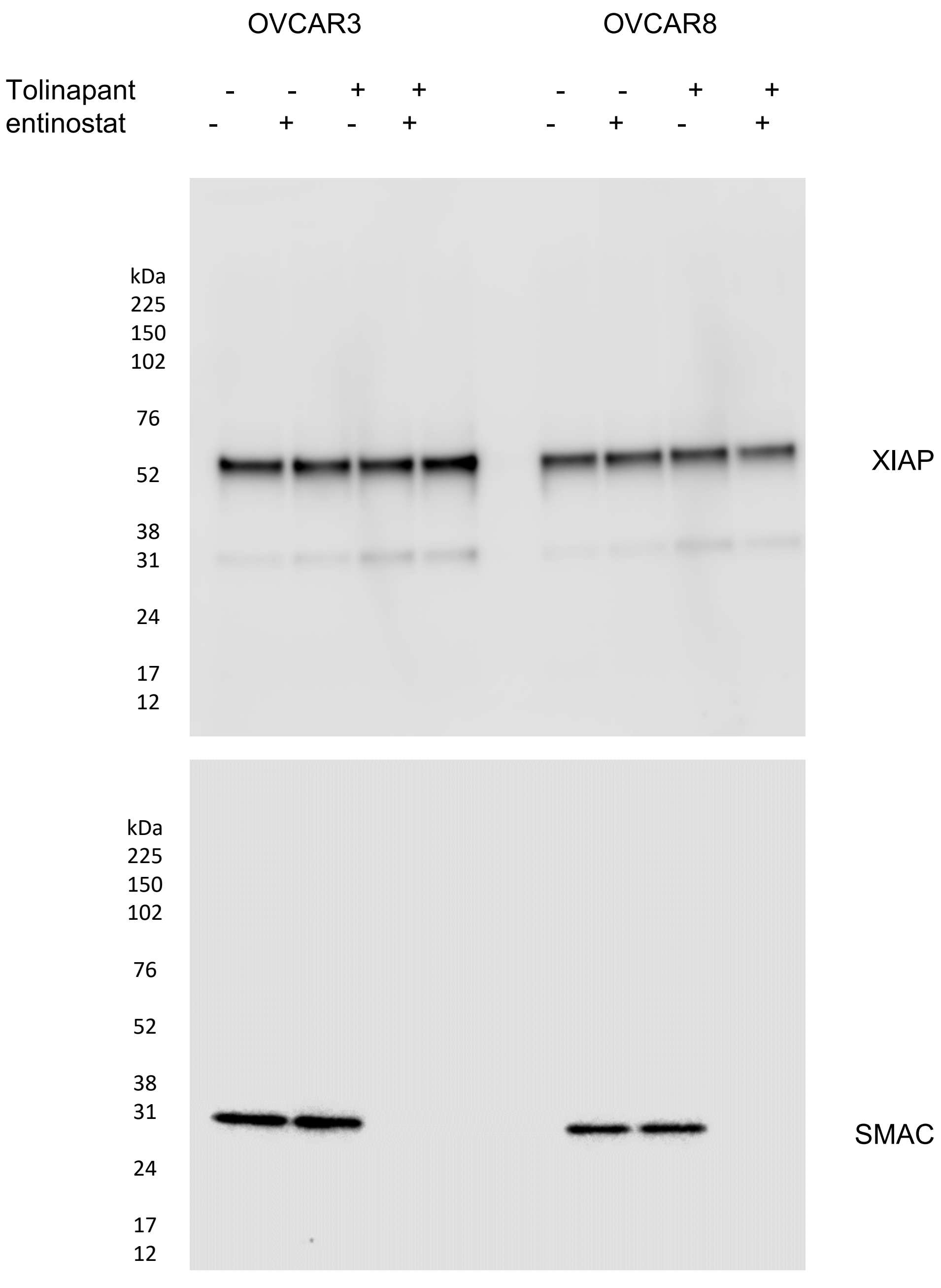

Supplemental Figure 7
Figure 3D whole blot

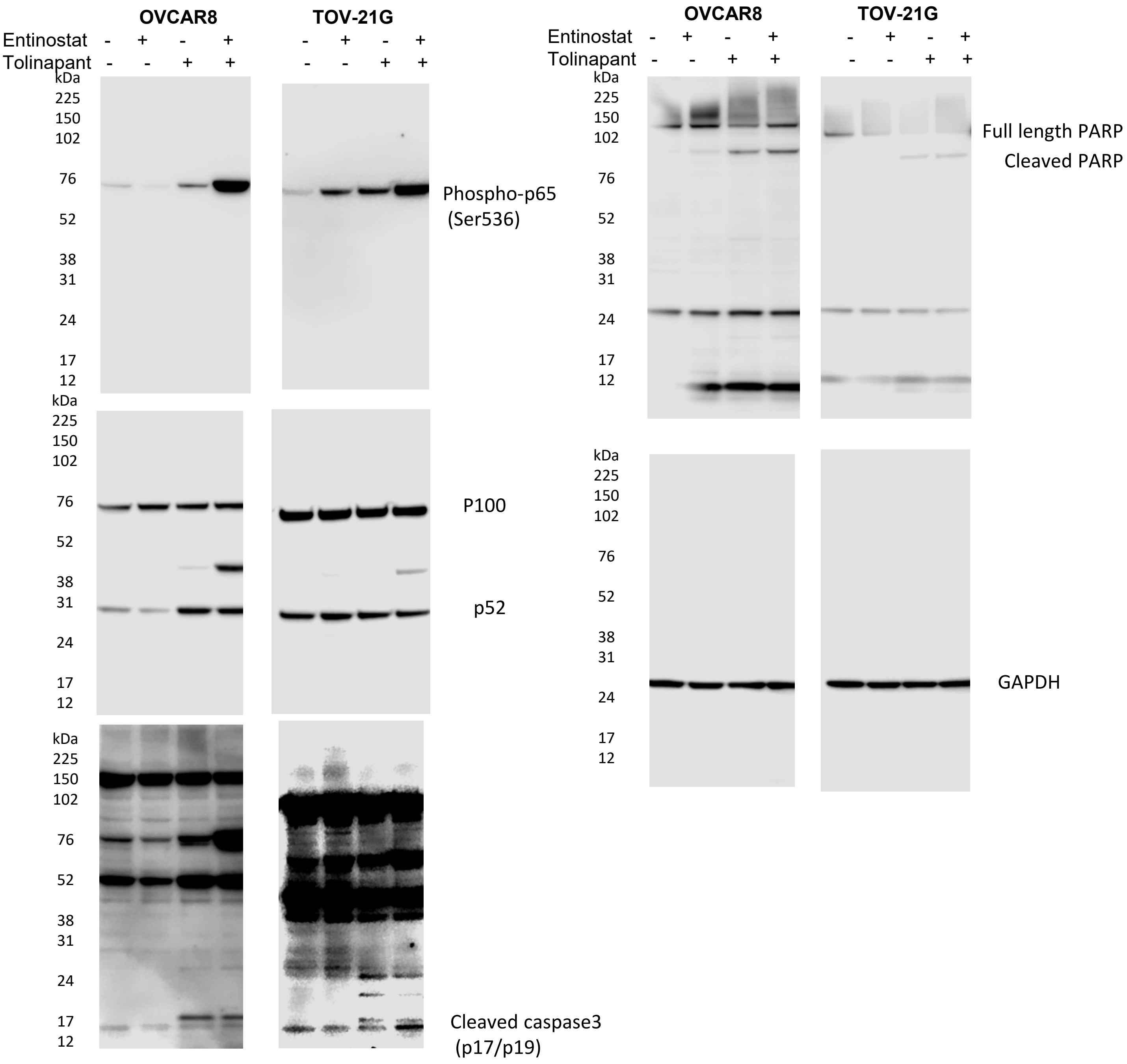

Supplement: Supplementary file 1 [file cancers-15-01315-s001.zip › Supplementary material/SMAC-HDAC_paper full blots.pdf]
