# Supplementary material for: SMAC Mimetics Synergistically Cooperate with HDAC Inhibitors Enhancing TNF-α Autocrine Signaling
Source: Cancers (Basel). 2023 Feb 18;15(4):1315. doi: 10.3390/cancers15041315 (PMC9954505; doi:10.3390/cancers15041315)
Supplement: Supplementary file 1 [file cancers-15-01315-s001.zip › Supplementary material/SMAC-HDAC_paper supple figures122222.pdf]

Supplemental Figure 8

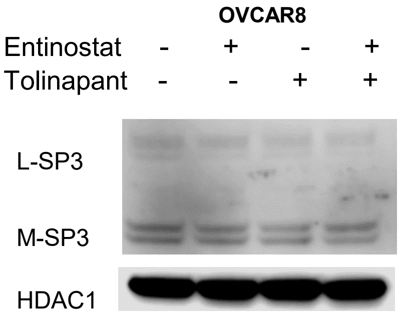

Supplemental Figure 9

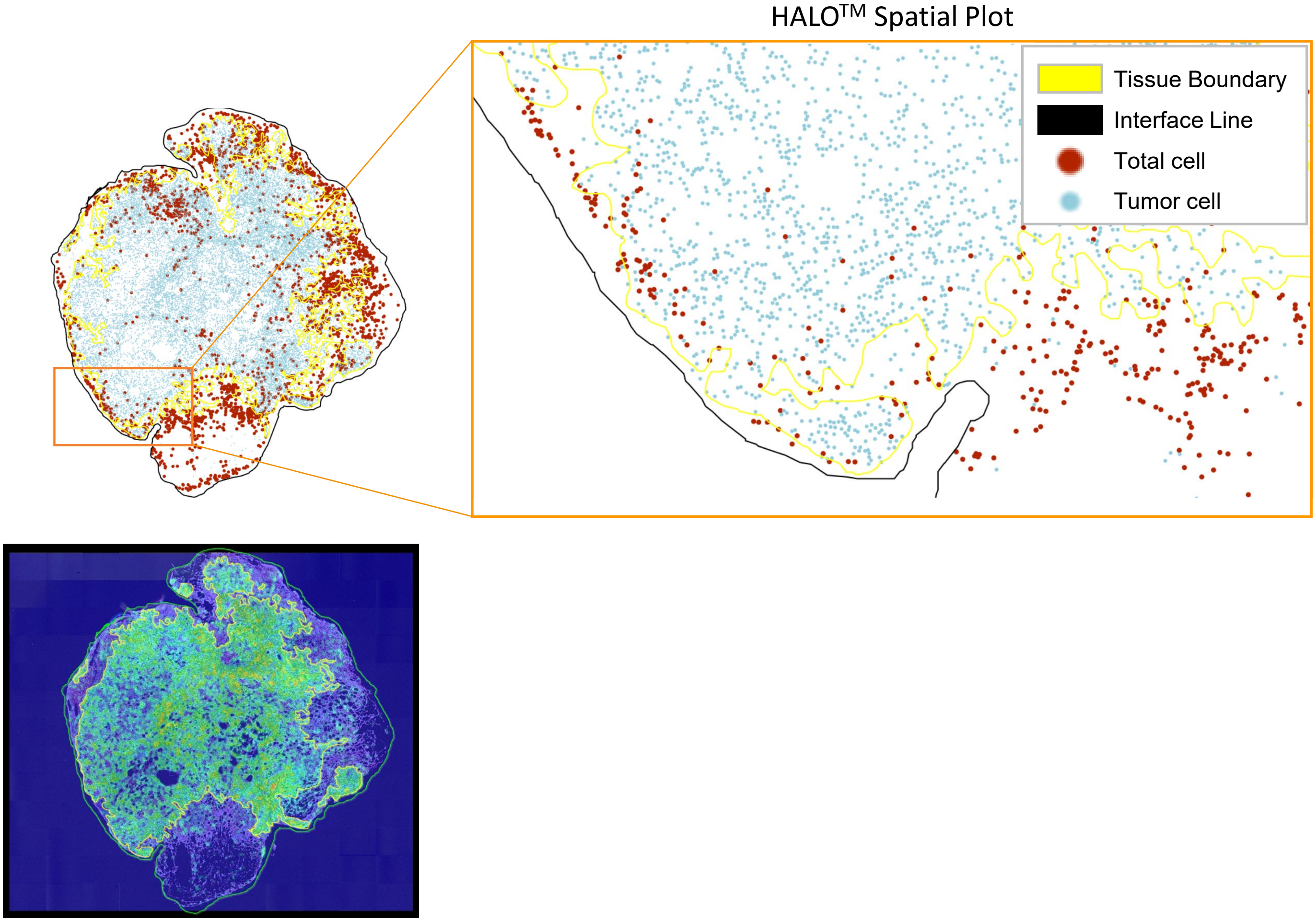

Supplemental Figure 10

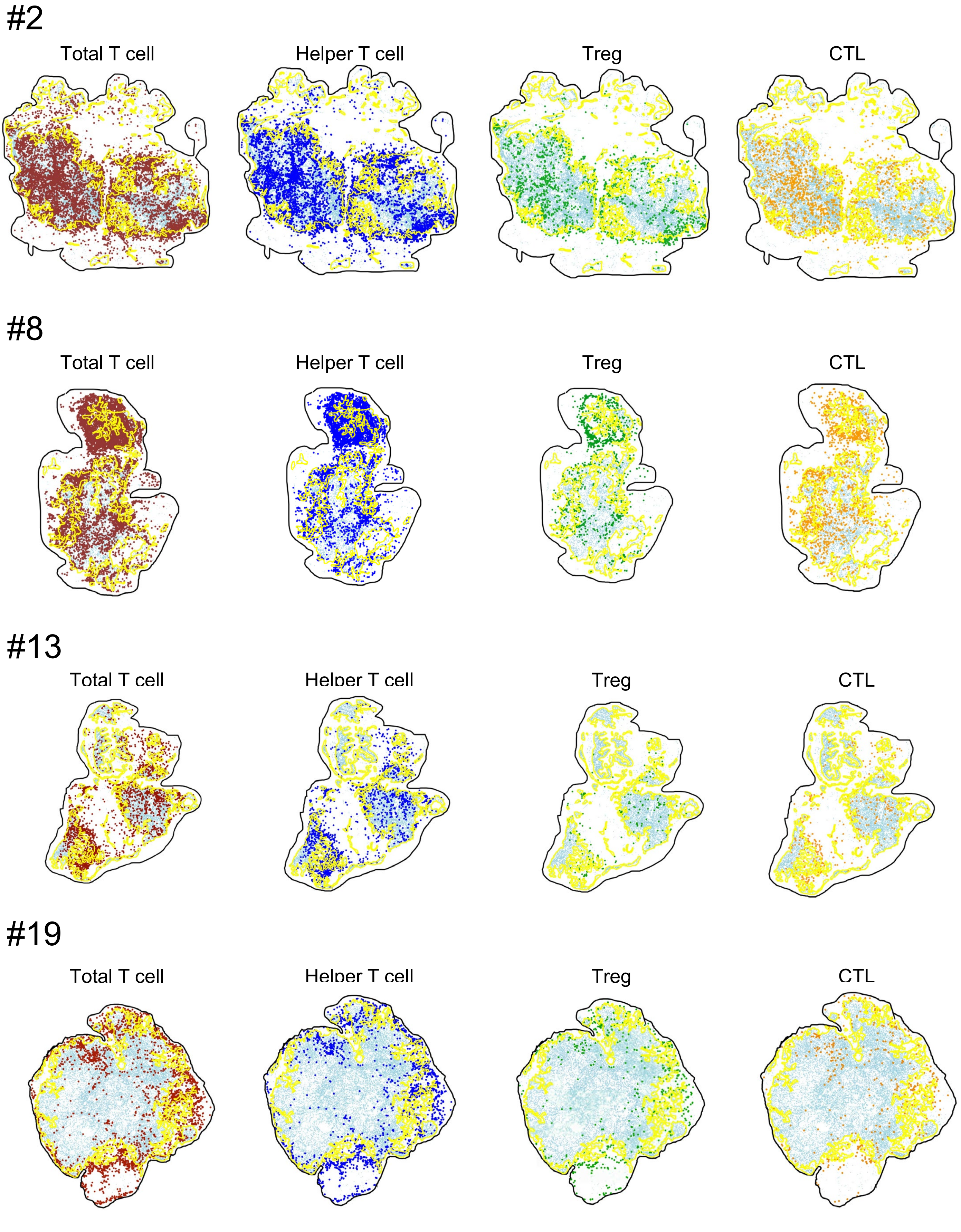

Supplemental Figure 11

			Log-rank test p value	Wilcoxon test p value
Vehicle + IgG	vs	ASTX660 + Entinostat + aPD1	0.001	0.0011
ASTX660 + IgG	vs	ASTX660 + Entinostat + aPD1	0.001	0.0011
Entinostat + IgG	vs	ASTX660 + Entinostat + aPD1	0.0401	0.0102
ASTX660 + Entinostat + IgG	vs	ASTX660 + Entinostat + aPD1	0.0029	0.0071
Vehicle + aPD1	vs	ASTX660 + Entinostat + aPD1	<0.001	0.001
ASTX660 + aPD1	vs	ASTX660 + Entinostat + aPD1	0.001	0.0011
Entinostat + aPD1	vs	ASTX660 + Entinostat + aPD1	0.001	0.0011
